# Skin hydrophobicity as an adaptation for self‐cleaning in geckos

**DOI:** 10.1002/ece3.6218

**Published:** 2020-04-12

**Authors:** Jendrian Riedel, Matthew John Vucko, Simone P. Blomberg, Lin Schwarzkopf

**Affiliations:** ^1^ College of Science and Engineering James Cook University Townsville Qld. Australia; ^2^ School of Biological Sciences University of Queensland St. Lucia Qld. Australia

**Keywords:** ancestral state reconstruction, contact angle, ecomorphology, evolution, Gekkota, hydrophobic surface properties, integument, phylogenetic generalized least squares

## Abstract

Hydrophobicity is common in plants and animals, typically caused by high relief microtexture functioning to keep the surface clean. Although the occurrence and physical causes of hydrophobicity are well understood, ecological factors promoting its evolution are unclear. Geckos have highly hydrophobic integuments. We predicted that, because the ground is dirty and filled with pathogens, high hydrophobicity should coevolve with terrestrial microhabitat use. Advancing contact‐angle (ACA) measurements of water droplets were used to quantify hydrophobicity in 24 species of Australian gecko. We reconstructed the evolution of ACA values, in relation to microhabitat use of geckos. To determine the best set of structural characteristics associated with the evolution of hydrophobicity, we used linear models fitted using phylogenetic generalized least squares (PGLS), and then model averaging based on AIC_c_ values. All species were highly hydrophobic (ACA > 132.72°), but terrestrial species had significantly higher ACA values than arboreal ones. The evolution of longer spinules and smaller scales was correlated with high hydrophobicity. These results suggest that hydrophobicity has coevolved with terrestrial microhabitat use in Australian geckos *via* selection for long spinules and small scales, likely to keep their skin clean and prevent fouling and disease.

## Graphical abstract

Hydrophobicity is common in plants and animals, but ecological factors promoting its evolution are still unclear. Using advancing contact‐angle measurements, we test that high hydrophobicity has coevolved with terrestrial microhabitat use in geckos, as ground is dirty and filled with pathogens. Our results suggest that hydrophobicity has coevolved with terrestrial microhabitat use in Australian geckos via selection for long spinules and small scales, likely to keep their skin clean and prevent disease and fouling.

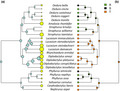

## 1 INTRODUCTION

The study of evolutionary morphology tackles questions examining how morphological traits may have evolved (Richter & Wirkner, 2014). Common study designs include mapping traits onto a phylogeny (Finarelli & Flynn, 2006; Hwang & Weirauch, 2012; King & Lee, 2015) or identifying correlations between traits and ecological factors (Kohlsdorf, Garland Jr, & Navas, 2001; Mihalitsis & Bellwood, 2019; Riedel, Vucko, Blomberg, Robson, & Schwarzkopf, 2019; Rothier, Brandt, & Kohlsdorf, 2017). Although these studies provide useful insights into the complex evolution of organisms and potential processes of adaptation, they only constitute the starting point of evolutionary research. Morphological traits often evolve to serve certain (and often multiple) functions, and therefore, the ecomorphology paradigm predicts that natural selection does not act on the morphological traits directly, but rather on their function (Garland Jr & Losos, 1994; Kluge, 1983). Functional morphological units often consist of complex multitrait structures, and there are multiple ways for a functionally complex organ to evolve and be optimized for particular outcomes, which can be conceptualized with many‐to‐one mapping (Alfaro, Bolnick, & Wainwright, 2005; Wainwright, 2007). However, if complex traits are analyzed as single traits, results can be misleading or difficult to interpret, so evolutionary morphology studies should address the evolution of functional outcomes directly (Garland Jr & Losos, 1994; Hagey et al., 2017; Russell & Gamble, 2019; Tiatragul, Murali, & Stroud, 2017).

The integument, which is the outermost layer of an organism, is a good example of a complex organ that serves multiple functions, the most important of which is protection from the surrounding environment (e.g., water loss, ultra‐violet radiation, pathogens, and predators). The integument can also be used for intra‐ or interspecific communication (Schliemann, 2015), locomotion (e.g., feathers in birds; Clarke, 2013; Homberger & de Silva, 2015; Maderson & Alibardi, 2000), or adhesion (e.g., in anoles or geckos; Maderson, 1964; Russell, 2002).

To ensure its functionality, the integument must be kept clean of dirt or debris, which may interfere with its functions. The ability to shed dirt is essential to prevent wear and tear (Irish, Williams, & Seling, 1988), to reduce the accumulation of excess weight, to avoid interfering with crypsis, signaling, or other specialized functions (Arnold, 2002; Gans & Baic, 1977; Hansen & Autumn, 2005), and to reduce exposure to pathogens (Neinhuis & Barthlott, 1997; Watson, Green, et al., 2015). Various functional and behavioral mechanisms have evolved to keep living surface structures clean and pathogen‐free. A common behavioral mechanism is grooming (i.e., Bauer, 1981; Sparks, 1967), and shedding may assist in dirt removal (Böhme & Fischer, 2000; Fushida, Riedel, Nordberg, Pillai, & Schwarzkopf, 2020). A common structural solution to fouling is increased surface hydrophobicity (Fusetani, 2004; Neinhuis & Barthlott, 1997; Wagner, Fürstner, Barthlott, & Neinhuis, 2003; Wagner, Neinhuis, & Barthlott, 1996). This “self‐cleaning” phenomenon, often termed the “lotus effect” (Barthlott & Neinhuis, 1997; Carbone & Mangialardi, 2005), helps to avoid fouling (Neinhuis & Barthlott, 1997; Watson, Cribb, Schwarzkopf, & Watson, 2015) or enable floating (Gao & Jiang, 2004; Perez‐Goodwyn, 2009) and occurs in plants, insects, and vertebrates (Hiller, 2009; Neinhuis & Barthlott, 1997; Spinner, Gorb, & Westhoff, 2013; Wagner et al., 2003).

Hydrophobic surfaces repel water *via* surface texture or chemistry, such that the forces within the water droplet are stronger than those attracting the water to the surface; therefore, water beads on hydrophobic surfaces (Cassie & Baxter, 1944). A surface is defined as hydrophobic if the contact angle of a drop of water placed on that surface is greater than 90°, and superhydrophobic if the contact angle is greater than 150° (Li, Reinhoudt, & Crego‐Calama, 2007). In structural hydrophobicity, hydrophobic properties are enhanced by increasing surface roughness, a product of complex hierarchical microstructures, which greatly reduce the contact angles of water droplets (Barthlott & Neinhuis, 1997; Cassie & Baxter, 1944; Shah & Sitti, 2004).

The occurrence and function of hydrophobicity have been well studied in plants (Neinhuis & Barthlott, 1997, 1998; Wagner et al., 2003) and insects (Byun et al., 2009; Voigt, Boeve, & Gorb, 2012; Watson et al., 2010). For example, the wings of insects are covered with self‐cleaning microstructures, thought to maintain flying ability (Wagner et al., 1996; Watson et al., 2010), and in plants, hydrophobic self‐cleaning surfaces may protect against harmful microorganisms, which are growth‐inhibited by the dry plant surface (Neinhuis & Barthlott, 1997). In some plants (e.g., water fern [Salvinia]), hydrophobic leaf surfaces ensure efficient gas exchange by keeping a thin film of air clinging to the surface when the leaves are submerged (Cerman, Striffler, & Barthlott, 2009). Underwater breathing has also evolved in insects (especially Heteroptera) and spiders, where some species associated with water form a thin layer of air (plastron) around the body (Perez‐Goodwyn, 2009; Stratton & Suter, 2009), and potentially also in some *Anolis* lizards (Swierk, 2019). Another function of hydrophobic skin surfaces is to prevent submersion, allowing animals to live on or at the water surface (e.g., water striders (Gerromorpha, Heteroptera; Gao & Jiang, 2004) and spiders (Stratton & Suter, 2009)). In contrast, the hydrophobic properties of the integument of vertebrates have not been well documented (but see Hiller, 2009; Nirody et al., 2018; Stark & Mitchell, 2019; Watson, Green, et al., 2015). More importantly, while the physical principles and function of hydrophobic surfaces are well understood (Li et al., 2007), it is unclear which selective pressures promote the evolution of this functional adaptation.

Our study aimed to analyze the evolution of hydrophobicity, specifically hydrophobic surface characteristics, using geckos (Gekkota) as a model system. Geckos are an ideal model in that they are a successful, species‐rich clade with a worldwide distribution (Meiri, 2020; Uetz & Jirí Hošek, 2019), and their skin surfaces are hydrophobic because of small microstructures (spinules) covering the outermost layer of the epidermis (Hiller, 2009; Ruibal, 1968). Recent studies have demonstrated that these microstructures are not only hydrophobic and lead to self‐cleaning, but spinules are also bactericidal, making them a good example of multifunctional morphological traits (Watson, Cribb, et al., 2015; Watson, Green, et al., 2015). This bactericidal quality occurs because larger bacteria are pierced by the spinules and smaller bacteria get damaged by stretching and tearing between spinules (Li et al., 2016; Watson, Cribb, et al., 2015).

If hydrophobicity has evolved to promote self‐cleaning and bactericidal functions in geckos, we expect that terrestrial species would be better adapted for self‐cleaning and possess better bactericidal skin properties than species using other habitats, as dust, dirt, debris, and bacteria tend to accumulate on the ground (McCabe, Reader, & Nunn, 2015; Nunn, Gittleman, & Antonovics, 2000; Ungar, Teaford, Glander, & Pastor, 1995). Conversely, as microbial growth rates are rapid in humid rainforest habitats, compared with more arid environments, and as high humidity enhances soiling because water facilitates the spread of dirt particles, hydrophobic and bactericidal skin properties may also be more prominent in species from habitats with a higher average humidity (Arnold, 2002; Bouskill et al., 2012).

A recent study of Australian geckos found that long, dense spinules, in combination with small scales, were associated with terrestrial microhabitats, but not with a high habitat humidity (Riedel et al., 2019). According to the ecomorphology paradigm, trait selection should operate on the function of a trait, in this case, on its hydrophobic, self‐cleaning, and bactericidal properties, instead of directly on the morphology of the traits, scale size and spinule length, density, or morphology. Therefore, if the association between small scales and long and dense spinules is truly an adaptation to terrestrial microhabitats, these morphological traits should affect the proposed function (hydrophobicity), which should in turn be the target of natural selection. Subsequently, this selection should be reflected in the evolutionary history of geckos (Garland Jr & Losos, 1994; Kluge, 1983). Thus, we would expect terrestrial gecko species to evolve hydrophobicity at greater rates than those of arboreal or saxicoline (rock‐dwelling) gecko species, and that hydrophobicity has evolved in association with terrestrial microhabitat use. Using the same reasoning, species occupying habitats with high average humidity (e.g., rainforests) may also select for higher hydrophobicity compared with those from drier habitats (e.g., savannahs or deserts).

In this study, we examined the evolution of hydrophobicity of gecko skin, using the Diplodactyloid families Diplodactylidae and Carphodactylidae. The sessile drop technique (Drelich, 2013; Kwok, Gietzelt, Grundke, Jacobasch, & Neumann, 1997) was modified for use on living animals to quantify advancing contact angles (ACA) on 24 species, for which detailed measurements of skin microornamentation have been made previously (Riedel et al., 2019). We analyzed the correlation of ACA values with both microhabitat use and habitat humidity in a phylogenetically informed context, using a modified version of a published phylogeny from Brennan and Oliver (2017). In addition, we reconstructed the ancestral states of the ACA values and microhabitat use and habitat humidity to test whether these traits have evolved together. We predicted that (a) hydrophobicity has evolved together with terrestrial microhabitat use in geckos and may be associated with habitats that have high humidity, and (b) hydrophobicity is primarily driven by relatively dense, long spinules and relatively small scale size. Both predictions are consistent with the hypothesis that the hydrophobic integument of Australian geckos has evolved as an adaptation to promote self‐cleaning and bactericidal properties in a terrestrial microhabitat.

## 2 MATERIAL AND METHODS

### 2.1 Study species

Specimens of 24 species of Australian Carphodactylidae (6) and Diplodactylidae (18) were collected at night by hand, and habitat humidity (hydric, mesic, and xeric) (Bureau of Meteorology, 2018) and microhabitat use (terrestrial, arboreal, and saxicoline) were assigned to each species (Table 1) in the latter case using both data recorded at the time of collection and the literature (Cogger, 2015; Meiri, 2018; Nordberg & Schwarzkopf, 2019). We classified white‐striped geckos (*Strophurus taeniatus*) as “terrestrial” for this analysis as this species is strongly associated with spinifex hummock or porcupine grass (*Triodia *spp.; Laver, Nielsen, Rosauer, & Oliver, 2017; Nielsen, Oliver, Laver, Bauer, & Noonan, 2016), which is a very low growing plant, and in our study, *S. taeniatus* was always found close to, or on, the ground (pers. obs.). After collection, healthy adult specimens were brought back to the laboratory to quantify hydrophobic properties. Microornamentation measurements were taken from Riedel et al. (2019), which were obtained from the same specimens used for the ACA measurements.

**Table 1:** Microhabitat use, habitat humidity, and mean (± *SE*) advancing contact‐angle (ACA) measurements for each species. The number of specimens measured for each species is given (*N*_S_), as well as the mean number of measurements for each individual per species (*N*_I_)

Species	Habitat	Humidity	ACA (°)	*N*_S_	*N*_I_
Carphodactylidae					
*Carphodactylus laevis*	Terrestrial	Hydric	144.49 ± 0.67	2	14
*Nephrurus asper*	Terrestrial	Mesic	133.94 ± 0.33	11	12
*Phyllurus amnicola*	Saxicoline	Hydric	143.40 ± 0.71	1	16
*Phyllurus nepthys*	Arboreal	Hydric	137.43 ± 0.86	3	12
*Phyllurus ossa*	Saxicoline	Hydric	138.32 ± 0.53	5	18
*Saltuarius cornutus*	Arboreal	Hydric	136.02 ± 0.32	13	10
Diplodactylidae					
*Amalosia rhombifer*	Arboreal	Mesic	150.63 ± 0.70	6	13
*Diplodactylus ameyi*	Terrestrial	Xeric	152.60 ± 0.47	3	8
*Diplodactylus conspicillatus*	Terrestrial	Xeric	149.57 ± 0.80	2	6
*Diplodactylus platyurus*	Terrestrial	Mesic	146.81 ± 0.40	12	11
*Diplodactylus tessellatus*	Terrestrial	Xeric	147.63 ± 0.57	12	9
*Lucasium damaeum*	Terrestrial	Xeric	155.58 ± 0.52	2	7
*Lucasium immaculatum*	Terrestrial	Xeric	140.32 ± 0.64	4	13
*Lucasium steindachneri*	Terrestrial	Mesic	150.45 ± 0.39	23	8
*Lucasium stenodactylum*	Terrestrial	Xeric	141.16 ± 0.61	2	14
*Oedura bella*	Saxicoline	Xeric	140.04 ± 0.79	1	16
*Oedura castelnaui*	Arboreal	Mesic	134.66 ± 0.41	9	12
*Oedura cincta*	Arboreal	Xeric	133.71 ± 0.41	9	14
*Oedura coggeri*	Saxicoline	Mesic	136.82 ± 0.38	7	14
*Oedura monilis*	Arboreal	Mesic	140.82 ± 0.49	15	10
*Rhynchoedura ormsbyi*	Terrestrial	Xeric	147.23 ± 1.07	7	8
*Strophurus krisalys*	Arboreal	Xeric	130.72 ± 0.45	16	9
*Strophurus taeniatus*	Terrestrial	Mesic	152.43 ± 1.14	1	4
*Strophurus williamsi*	Arboreal	Mesic	136.23 ± 0.37	19	10

### 2.2 Advancing contact‐angle measurements

Increases in ACA of water droplets on surfaces are correlated with increasing hydrophobic properties of that surface (Li et al., 2007). Therefore, the sessile drop technique was used to quantify contact angles of droplets of distilled water, incrementally increased in size (see below), and photographed at high resolution. The contact angles could then be used to predict the hydrophobicity of the dorsal skin surface (Li et al., 2007). All contact‐angle measurements were carried out under laboratory conditions at room temperature (23°C) between four and eight days after each individual shed its skin.

Measurements were only successful if the animals remained completely still and, to ensure this, their fore and hind limbs were outstretched and taped to the body using 3M Micropore™ tape, which was easily removable, leaving no residue. Limb immobilization was accomplished without touching the dorsal scales to avoid affecting any dorsal scale microornamentation, or leaving chemical residues from fingers or tape. In addition to being restrained, it was necessary for each individual to present a flat surface for the accurate measurement of contact angles. To compensate for vertebral column unevenness and motion due to breathing, each lizard was rolled slightly to one side, to ensure that the area just lateral to the vertebral column was level. Movement due to breathing was mitigated by measuring contact angles toward the posterior of the torso on a flat area, and within the area assessed for skin microornamentation in Riedel et al. (2019). If breathing movements affected the entire length of the body, tape was placed lengthwise along each side of the lizard for a short period (30 s maximum), minimizing breathing movements, without affecting the well‐being of the lizard.

**Figure 1:**
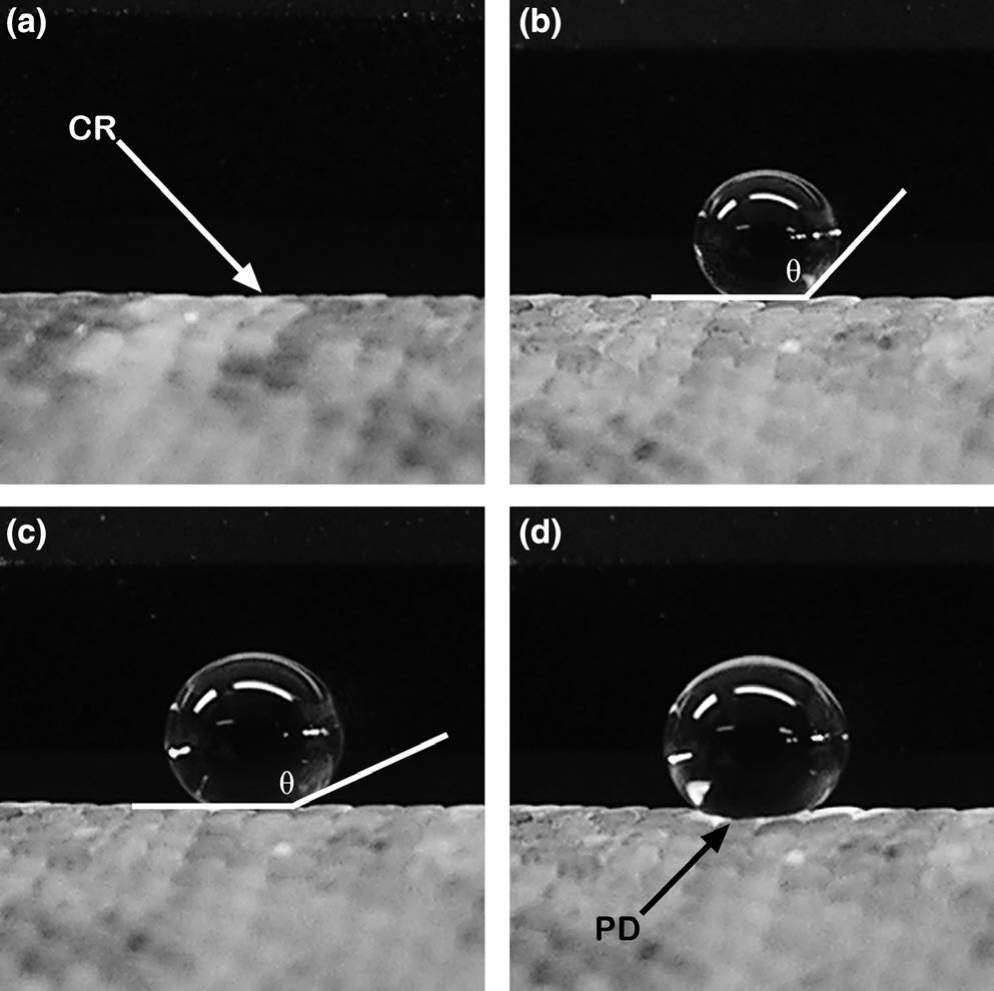
Photographic sequence of a “growing” drop used to obtain advancing contact‐angle (ACA) measurements on the dorsal scales of geckos. (a) Initial image of dorsal surface with the exact location where the scales crested (CR). (b) Initial, sessile drop placed on the dorsal surface with a contact angle (θ). (c) Droplet just previous to that which “popped out” (d), where θ represents the ACA that was obtained by adding 0.25 µl of distilled water to the initial water droplet (b) numerous times. (d) One increment after the ACA measurement (c) in which the droplet had popped out (PD) and, in this case, projected toward the camera

Once the lizard was immobilized and a suitable, level area on the dorsal surface was available (Figure 1a), ACA was quantified. Droplets were placed onto the lizard's body using an Eppendorf® pipette with a volume capacity of 0.1–2.5 µl and expanded slowly by adding water to the droplet in increments of 0.25 µl (Figure 1b). The initial, sessile drop was placed at the point at which the scales crested (Figure 1a), and the ACA values were attained by adding 0.25 µl to the sessile water droplet numerous times (Figure 1c). The expanding water droplet was photographed at each 0.25 µl increment, until the base of the drop appeared to “pop” out to the side or the droplet started to roll (Figure 1d). The ACA was measured on the photograph taken immediately before the drop “popped out” or rolled (Figure 1c). Although very difficult to see with the naked eye, on enlarged photographs ACA was clearly visible. Typically, less than 12 increments of 0.25 µl (3 µl) or 12 photographs were required to produce a drop suitable for an ACA measurement. Angles were measured using ImageJ (v. 1.36b, Schneider, Rasband, & Eliceiri, 2012), and median contact angles were calculated for each individual (individuals within each species were measured between 4 and 18 times on average; see Table 1) and used to calculate means for each species.

### 2.3 Statistical analysis of ecology and hydrophobicity

Phylogenetic generalized least square analysis (PLGS) (Martins & Hansen, 1997; Revell & Collar, 2009) was used to examine the association between hydrophobicity (ACA measurements), and the microhabitat (terrestrial, arboreal, and saxicoline) and habitat humidity (hydric, mesic, and xeric) used by geckos, respectively. A modified version of the phylogenetic tree published by Brennan and Oliver (2017) was used, which included all the species from this study except for *Phyllurus nepthys*, for which no genetic sample was available. Therefore, we replaced *P. championae* on the tree with *P. nepthys*, as it is the closest relative for which data were available (Hoskin & Couper, 2013; C.J. Hoskin, pers. com.). The PGLS models were fitted comparing three different models of trait evolution, Brownian motion (λ = 1; “BM”; Felsenstein, 1985), a relaxed maximum‐likelihood value of λ (“Pagel”), and without any phylogenetic signal in the trait evolution (“star model,” λ set to 0; Pagel, 1992). The ACA values were the response variable, and microhabitat and humidity were explanatory variables (both as fixed effects, combined in each model without an interaction term). Model averaging was then used (Bumham & Anderson, 2002), based on AIC_c_, to combine the inferences from all valid models (AIC_c_ < 2) and type II ANOVAs (Langsrud, 2003), followed by a Tukey's *post hoc* test using the R package “emmeans” (R Core Team, 2018; Russell, 2018) to determine significant differences.

The ancestral states of ACA values were constructed using the “ace” function from the “ape” package in R (Paradis, Claude, & Strimmer, 2004), and complemented with an ancestral state reconstruction of the explanatory variable(s). A "symmetrical" model was then fitted, which constrained forward transitions to be equivalent to backward transitions, as preliminary analysis demonstrated that there were not enough transitions to estimate a more complicated model (Pagel, 1999; Schluter, Price, Mooers, & Ludwig, 1997).

### 2.4 Statistical analysis of morphology and performance

The morphological dataset from Riedel et al. (2019) was used to analyze the influence of microornamentation and skin characteristics (both log‐transformed) on ACA values, but excluded all cutaneous sensilla measurements, as cutaneous sensilla only covered a minute proportion of the skin surface and were unlikely to have a large effect on hydrophobicity. Therefore, seven measurements for microornamentation were used: spinule length (SL), spinule density (*SD*), diameter (PDM) and density (PDE) of the pits (small indentations between the spinules), the percentage of area covered by knobs (KI), and two measures of scale size (granule size [GS] and intergranule size [IGS]). Two sets of PGLS models were then constructed, one including all seven of these morphological traits (M1), and one containing only those traits that contributed strongly to separate the species by microhabitat (spinule length, pit diameter, knobiness, granule size, and intergranule size) in Riedel et al. (2019) (M2). To ensure the validity of these models, all traits were tested for multicollinearity (Mundry, 2014). As the scale size measurements for both types of scales (GS and IGS) are strongly correlated (*r* (22) = 0.86; *p* < 0.001), the abovementioned models were constructed twice, once containing only GS (M1G and M2G) and once containing only IGS (M1I and M2I). This produced four models with specific sets of predictor variables. Otherwise, the models were constructed using the same approach already described (see “Statistical analysis of ecology and hydrophobicity”). Notably, as the optimal λ value was inside a biologically meaningful range of 0 to 1 only for the M2I model, the optimal λ model was not fitted or used for all other models in the set of candidate models.

## 3 RESULTS

### 3.1 Advancing contact‐angle measurements

All gecko species examined were hydrophobic, in that they had contact angles above 90°, but some species were more hydrophobic than others, including several that were superhydrophobic, with mean contact angles above 150° (Table 1). These included *L. steindachneri* (150.45 ± 0.39°), *A. rhombifer* (150.63 ± 0.70°), *S. taeniatus* (152.43 ± 1.14°), *D. conspicillatus* (152.60 ± 0.47°), and *L. damaeum* (155.58 ± 0.52°).

### 3.2 Correlation between hydrophobicity and microhabitat

**Figure 2:**
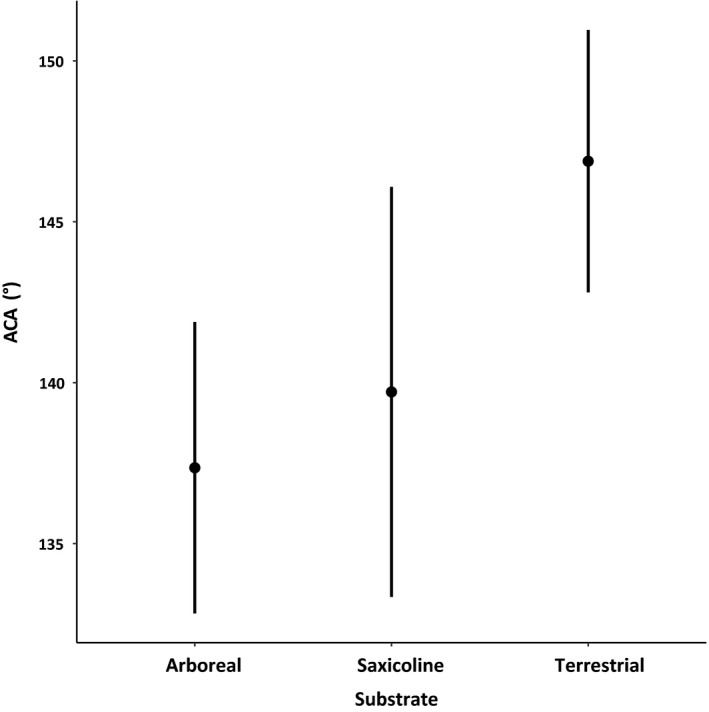
Effects plot of the best model (Star model with *λ* = 0) of the ACA values grouped by microhabitat use. Terrestrial species have significantly higher ACA values than arboreal species, with the saxicoline ones falling in between both

**Figure 3:**
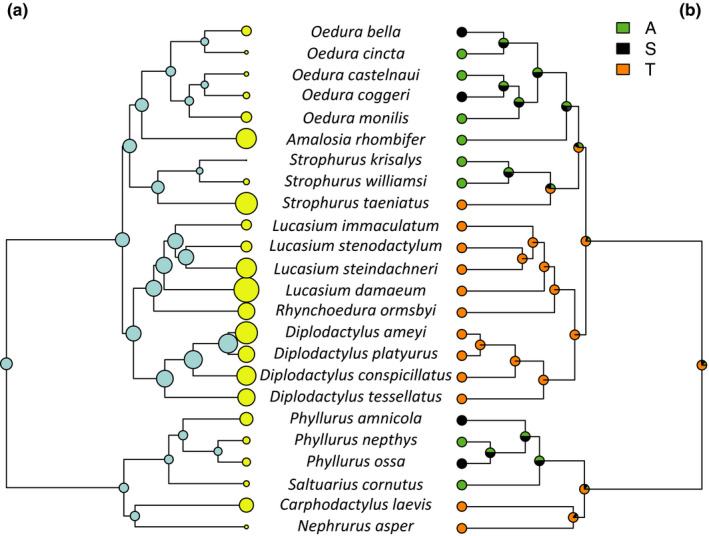
Reconstructed ancestral states of hydrophobicity (a) and microhabitat use (b). For hydrophobicity, the size of the dots correlates with the ACA measurements for the species of this study (yellow dots) and reconstructed for the nodes (blue dots). For microhabitat use, the dots correspond to the reconstructed probability of microhabitat use for nodes: A (green), arboreal; S (black), saxicoline; T (orange), terrestrial. Note the correspondence between brown nodes (terrestrial species) and large yellow circles (hydrophobic species)

**Figure 4:**
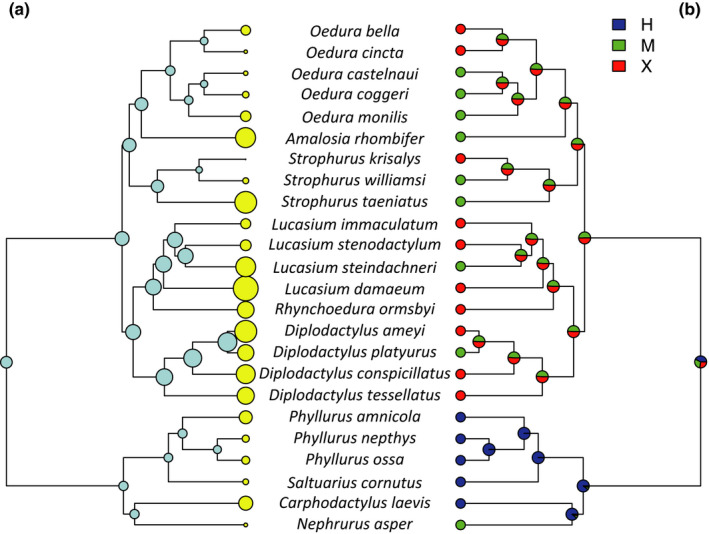
Reconstructed ancestral states of hydrophobicity (a) and habitat humidity (b). The hydrophobicity reconstruction is identical to Figure 3a. For habitat humidity, the dots correspond to the reconstructed probability of microhabitat use for nodes: H (blue), humid (rainforest); M (green), mesic (savanna); X (orange), xeric (desert). Neither high nor low hydrophobic species correspond with a particular habitat humidity regime

There was a significant effect of microhabitat on ACA in all models, such that terrestrial species had higher ACA values than arboreal ones, and there were no significant differences between the saxicoline species and the other two groups (Table 2B; Figure 2). Habitat humidity did not have a significant effect on ACA in any of the models (Table 2B). The optimal λ value of the Pagel model was 0.85, and both the Star model and the Pagel model had high support values (ΔAIC_c_ 0.00 and 0.36, respectively). However, the BM model also had reasonable support just outside the cutoff (Delta AIC_c_ = 2.01; Table 2A); therefore, an ANOVA (type II) was used to determine significance of terms for all evolutionary models. The reconstructed ancestral states of the analyzed traits are presented in Figures 3 and 4. The reconstructed evolution of the ACA values was reflected in the evolution of microhabitat use, and both traits had a similar evolutionary history (Figure 3). Notable exceptions from this pattern are *Amalosia rhombifer*, which had a high ACA (150.63 ± 0.70°) despite being arboreal, and *Nephrurus asper*, which had a relatively low ACA (133.94 ± 0.33°) despite being terrestrial. The reconstructed evolution of habitat humidity use did not show any correlation with the evolution of ACA values (Figure 4).

**Table 2:** (A) Results of the model selection for the different modes of trait evolution to test whether hydrophobicity (ACA measurements) could be explained by either microhabitat use (substrate) or habitat humidity. (B) *p*‐values for each of the two explanatory variables for each model and results of a post hoc pairwise comparisons for the significant variables

Mode of trait evolution	*df*	log Likelihood	AIC_C_	ΔAIC	AICc weight	Cumulated weight
(A) model comparison						
Star	6	−74.23	165.39	0	0.45	0.45
Pagel (*λ* = 0.85)	6	−74.41	165.76	0.36	0.38	0.83
BM	6	−75.23	167.41	2.01	0.17	1

Explanatory variable	*df*	*χ* ^2^	*p*	Tukey's *Post hoc* pairwise comparison
(B) ANOVA results				
Star model				
Substrate	2	11.473	**0.003**	**Terrestrial > Arboreal**
Habitat humidity	2	0.164	0.921	
Pagel model				
Substrate	2	8.016	**0.018**	**Terrestrial > Arboreal**
Habitat humidity	2	1.912	0.384	
BM model				
Substrate	2	6.369	**0.041**	**Terrestrial > Arboreal**
Habitat humidity	2	1.316	0.518	

### 3.3 Correlation between hydrophobicity and morphology

Pagel's *λ* was only estimable (*λ* = 0.13) for the M2I model, which included the subset of traits and IGS. *λ* was negative for all other models, indicating a poor fit of the lambda model to the data. Thus, only the BM and Star models were used for all other trait combinations, producing nine estimate models (Table 3). The model comparison revealed two models with ΔAIC_C_ < 2, namely the two variants of the M2 model, which included only the traits that were strongly correlated with microhabitat use in Riedel et al. (2019), and no phylogenetic signal in trait evolution assumed by the models (M2G.Star and M2I.Star; Table 3).

**Table 3:** Model comparison for the morphological traits sorted by ΔAIC values. The two models with high support are highlighted in bold. Morphological traits (explanatory variables) are spinule length (SL), spinule density (*SD*), pit diameter (PDM), pit density (PDE), granule scale size (GS), intergranule scale size (IGS), and percentage of area covered by knobs (KI)

Model	Explanatory variables	λ	*df*	Log likelihood	AIC_C_	ΔAIC	AICc weight	Cum. weight
**M2G.Star**	**SL, PDM, KI, GS**	0	6	−72.01	160.97	**0**	0.57	0.57
**M2I.Star**	**SL, PDM; KI, IGS**	0	6	−72.99	162.91	**1.95**	0.22	0.79
M2I.Pagel	SL, PDM, KI, IGS	0.13	6	−73.59	164.12	3.16	0.12	0.9
M1G.Star	SL, SD, PDM, PDE, GS, KI	0	8	−70.48	166.56	5.59	0.03	0.94
M2G.BM	SL, PDM, KI, GS	1	6	−74.89	166.71	5.75	0.03	0.97
M2I.BM	SL, PDM, KI, IGS	1	6	−75.39	167.72	6.76	0.02	0.99
M1I.Star	SL, SD, PDM, PDE, IGS, KI	0	8	−71.86	169.31	8.35	0.01	1
M1G.BM	SL, SD, PDM, PDE, GS, KI	1	8	−74.31	174.21	13.25	0	1
M1I.BM	SL, SD, PDM, PDE, IGS, KI	1	8	−75.1	175.8	14.81	0	1

In both models, spinule length was significantly positively associated with ACA values, whereas the scale size trait (GS or IGS) included in each model was significantly negatively associated with ACA measurements. In the second‐best model, which used IGS, pit diameter was also significantly negatively associated with the ACA measurements (Table 4).

**Table 4:** Predictors (morphological traits) in the two models with the highest support. Significant *p*‐values are highlighted in bold, and coefficients represent a positive or negative correlation with the ACA measurements

Trait	M2G.Star				M2I.Star			
	coefficient	*p*‐Value	*χ* ^2^	*df*	coefficient	*p*‐Value	*χ* ^2^	*df*
Spinule length	49.24	**0.007**	7.09	1	49.24	**0.039**	4.23	1
Granule size	−84.44	**0.006**	7.52	1	NA	NA	NA	NA
Intergranule size	NA	NA	NA	NA	−912.39	**0.019**	5.45	1
Pit diameter	−99.31	0.25	1.32	1	−99.31	**0.049**	3.89	1
Knobbiness	−56.67	0.15	2.07	1	−56.67	0.161	1.97	1

## 4 DISCUSSION

The outcomes of our study were consistent with our first prediction, and the hydrophobicity of geckos using terrestrial microhabitats was higher than those using arboreal habitats (Figure 2). In addition, we found that hydrophobicity and terrestrial microhabitat use have coevolved (Figure 3). There was no support for the second part of our first prediction that species from habitats with high humidity (rainforest) would have more hydrophobic skin (Figure 4). Our second prediction that hydrophobicity would be driven primarily by relatively long spinules and small scale size was supported, such that longer spinules and smaller scale size were important predictors of higher hydrophobicity. Contrary to our prediction, spinule density had no effect on hydrophobicity, but pit diameter, which contributes to the spacing between spinules, was an important predictor in the model using intergranule size, though not in the model using granule size. These results support our hypothesis that the hydrophobic integument of diplodactylid and carphodactylid geckos has probably evolved as an adaptation to keep their surfaces clean of dirt and debris and to inhibit the growth of potentially harmful bacteria prevalent in a terrestrial environment. Notably, the microhabitat was apparently a stronger selective force than was habitat humidity (Riedel et al., 2019). Hydrophobicity is related to highly irregular microscopic surface structures (Koch, Bhushan, & Barthlott, 2008; Wagner et al., 2003) that, in the case of geckos, consist of small scales and long spinules (Hiller, 2009; Ruibal, 1968). Our study confirms the importance of small scales and long spinules for the hydrophobic, self‐cleaning, and bactericidal functions of gecko skin.

We developed a method to quantify the hydrophobic properties of lizard skin in living lizards for this study, and established that, while all geckos were hydrophobic, several species were superhydrophobic and that most of the superhydrophobic species were terrestrial (Table 1). Although working with living lizards prevented us from strictly controlling humidity and vapor‐level conditions at the time of measurement as was suggested by Drelich (2013), all measurements were conducted under stable laboratory conditions (23°C and ~50% relative humidity). Combined with relatively high numbers of repeated measures, we received measurements with standard errors within the range of those (1–3°) reported by Drelich (2013).

Although the physical and functional basis of biological surfaces with hydrophobic properties has been well documented (Autumn & Hansen, 2006; Carbone & Mangialardi, 2005; Neinhuis & Barthlott, 1997; Stark, Ohlemacher, Knight, & Niewiarowski, 2015; Stark, Palecek, et al., 2015; Zhai et al., 2006), there has been little attention to the evolution of hydrophobic integumental properties. Associations of hydrophobic microstructures with aquatic or semiaquatic microhabitats in Heteroptera (Perez‐Goodwyn, 2009) are typically made in descriptive studies not analyzed using evolutionary statistics or in a phylogenetic context. For plants, Tellechea‐Robles, Salazar Ceseña, Bullock, Cadena‐Nava, and Méndez‐Alonzo (2019) tested the hypothesis that plants from coastal wetlands would be more hydrophobic if they grew in areas that flooded regularly, compared with areas that stayed dry, but the hypothesis was not supported by their study. Therefore, this is the first study to successfully test the predictions from a hypothesis about when and under which circumstances hydrophobicity may have evolved in nature, and provides phylogenetic statistical support for the evolution of hydrophobic surfaces as an apparent adaptation to the ecological pressures of living on the ground.

### 4.1 Hydrophobicity in geckos and other squamate reptiles

The ACA measurements in this study were within the range measured by previous studies for geckos. The arboreal gecko *Phelsuma laticauda* has a relatively low ACA of 139° (Hiller, 2009), whereas the highly derived ground‐dwelling legless gecko *Lialis jicari* (Pygopodidae) was superhydrophobic with an ACA of 160° on body regions not modified for their snake‐like locomotion (Spinner et al., 2013). Both examples support our hypothesis that terrestrial gecko species should be more hydrophobic than arboreal species. Saxicoline species fall between arboreal and terrestrial species, overlapping with both. Saxicoline species live on rock walls and in crevices between boulders, which can range from a few centimeters to many meters above the ground, and thus, species can be nearly terrestrial to almost never terrestrial, with their hydrophobicity likely varying in relation to their habitat requirements. Although variation in hydrophobicity was highest within saxicoline species, both arboreal and terrestrial species also varied considerably in their measured ACA. This variation could be correlated with differences in the rate of exposure to particle contamination within different microhabitats of the same category. For example, some tree species (e.g., paperbarks *Melaleuca *spp.) tend to be more granular and flaky than some others (e.g., ironbarks *Eucalyptus* spp.), which may increase exposure to bark debris. Similarly, different soil types may lead to differences in exposure to dust particles. More detailed knowledge of microhabitat use and particle exposure is necessary to elucidate this.

Long spinules as an adaptation to terrestrial microhabitats are particularly interesting in conjunction with the evolution of adhesive toepads. Adhesive toepads are adaptations to climbing, used in arboreal or saxicoline microhabitats (Russell, 1972, 2002). Setae, the microstructures generating adhesion in toepads, are proposed to have evolved by elongation of spinules (Ernst & Ruibal, 1966; Ruibal & Ernst, 1965; Russell, Baskerville, Gamble, & Higham, 2015; Russell & Gamble, 2019). Therefore, hair‐like microstructures in geckos appear to be an example in which a change in a morphological structure, which has evolved as an adaptation to one microhabitat, leads to a change in the trajectory of the adaptative potential of the changed morphological structure. For example, the elongation of spinules as an adaptation to terrestrial microhabitats may contribute to the elongation of setae on the subdigital scales, which in turn may lead to an enhanced adaptive potential to occupy climbing (arboreal or saxicoline) microhabitats. The elongated setae on toepads are still highly hydrophobic, and the hydrophobic properties of the setae are important to maintain the adhesive properties of the toepads in geckos, especially for species from humid environments with high rainfall such as rainforests (Stark & Mitchell, 2019; Stark, Palecek, et al., 2015; Stark, Sullivan, & Niewiarowski, 2012). More details on the relationship between spinule length and setae length of geckos from a range of habitats are required to examine evolution or coevolution of these characters.

Geckos are not the only reptilian taxon featuring spinule‐covered integuments. They share this trait with anoles and chameleons, as well as with some clades of skinks, agamids, and iguanids (Peterson, 1984; Ruibal, 1968). Anoles are a prime model group for ecomorphological studies, but previous studies have focused on morphometrics, such as relative limb dimensions, among species occupying different arboreal niches (Irschick, Vitt, Zani, & Losos, 1997; Losos, 1990, 1992; Losos, Jackman, Larson, de Queiroz, & Rodríguez‐Schettino, 1998), and have not examined the role of spinules. Chameleons are not only primarily arboreal, but also have exclusively terrestrial species. Interestingly, the mostly terrestrial pygmy chameleons (*Brookesia*, *Palleon*, *Rhampholeon*, *Rieppeleon*) and the terrestrial Namib chameleon (*Chameleo namaquensis*) have evolved honeycomb microstructures instead of, or in addition to, spinules (Riedel, Böhme, Bleckmann, & Spinner, 2015). Unfortunately, no studies on the hydrophobic properties of the integument of these two clades are available.

### 4.2 Function of hydrophobicity in geckos

The proposed function of hydrophobic surfaces in nature is to keep the surface of the integument free of dirt and debris, which can seriously obstruct other skin functions (Barthlott & Neinhuis, 1997; Hansen & Autumn, 2005; Watson, Schwarzkopf, et al., 2015). For this self‐cleaning ability, the surface needs to be hydrophobic and also must exhibit low adhesion forces for dirt particles. The integument of box‐patterned geckos (*Lucasium steindachenri*) has extremely low adhesion of artificial fouling particles (Watson, Cribb, et al., 2015), consistent with the terrestrial microhabitat use of this species, and the high ACA measures found in the present study. This combination of high hydrophobicity and low adhesion of fouling particles results in efficient self‐cleaning properties (Watson, Schwarzkopf, et al., 2015). Additional studies comparing self‐cleaning and adhesion forces for dirt particles could further enhance our understanding of this functional link. The spinule‐covered integument of geckos also has bactericidal properties (Li et al., 2016; Watson, Green, et al., 2015). Because exposure to potentially harmful microorganisms is higher in a terrestrial microhabitat (McCabe et al., 2015; Nunn et al., 2000) and bacterial growth rates and thus prevalence of microorganisms may be higher in habitats featuring high humidity (Bouskill et al., 2012), bactericidal properties could be prevalent in both terrestrial microhabitats and habitats with high humidity (e.g., rainforests). We found support only for the former expectation.

Possibly, hydrophobicity in rainforest geckos prevents drowning. In some insects and spiders, hydrophobic integumental properties facilitate the prevention of submersion (Gao & Jiang, 2004; Stratton & Suter, 2009). Although geckos are normally not associated with aquatic ecosystems, some geckos have advanced swimming abilities due to their hydrophobic skin (Nirody et al., 2018). Possibly, hydrophobicity may have evolved as an adaptation for species that are regularly threatened by flooding of their habitat. Under this hypothesis, we would expect terrestrial species to be more hydrophobic than arboreal species, but we would also expect stronger hydrophobic properties in rainforest habitats due to higher rainfall and more regular flooding. We would also expect saxicoline rainforest species (e.g., *Phyllurus amnicola* or *P. ossa*) to be strongly hydrophobic as they occur on boulders alongside rainforest streams. Therefore, drowning prevention is a plausible function promoting the evolution of hydrophobicity in some terrestrial geckos, but our results do not support drowning prevention as the dominant cause, because in our study, geckos from habitats most likely to flood were not the most hydrophobic. Our results more clearly support self‐cleaning as the main adaptive purpose for hydrophobicity, because in our study, geckos from dusty environments were the most hydrophobic.

Another hypothetic function of hydrophobic surfaces could be to reduce evaporative chill caused by water accumulation on nonhydrophobic surfaces (Cowles, 1958). If this was the main function of hydrophobicity in geckos, we would expect species from habitats with higher average rainfall (like rainforests) to be more hydrophobic than species from drier habitats, and no difference among microhabitats (arboreal, saxicoline, terrestrial). As the opposite signal was found in our study, this hypothesis was not supported by our results.

## CONFLICT OF INTEREST

The authors declare no conflict of interest.

## AUTHOR CONTRIBUTION

**Nils Jendrian Riedel:** Conceptualization (equal); Formal analysis (lead); Investigation (lead); Writing‐original draft (lead); Writing‐review & editing (lead). **Matthew John Vucko:** Conceptualization (equal); Formal analysis (supporting); Investigation (lead); Writing‐original draft (supporting); Writing‐review & editing (supporting). **Simone P. Blomberg:** Conceptualization (equal); Formal analysis (lead); Investigation (supporting); Writing‐original draft (supporting); Writing‐review & editing (supporting). **Lin Schwarzkopf:** Conceptualization (equal); Formal analysis (supporting); Investigation (lead); Supervision (lead); Writing‐original draft (supporting); Writing‐review & editing (supporting). 

## ETHICAL APPROVAL

Specimens were collected under the Scientific Purpose Permit No. WISP03476106 (Queensland Department of Environment and Heritage Protection, QLD, Australia), the Natural Resources Permit No. WITK03036906 (Department of National Parks, Sports, and Racing for protected areas, QLD, Australia), and the Permit to Undertake Scientific Research No. C25337 (Department of Environment and Water, South Australia, Australia). All work was carried out under the James Cook University Animal Ethics Approval A997.

## Data Availability

A summary of the measurements (mean and *SD* of the ACA values for each species) is given in the manuscript. Detailed measurements for each specimen are available from the corresponding author on reasonable request and were stored at Dryad under https://doi.org/10.5061/dryad.xwdbrv19s.
